# Author Correction: Genome-wide association study identifies susceptibility loci for B-cell childhood acute lymphoblastic leukemia

**DOI:** 10.1038/s41467-018-08106-9

**Published:** 2019-01-21

**Authors:** Jayaram Vijayakrishnan, James Studd, Peter Broderick, Ben Kinnersley, Amy Holroyd, Philip J. Law, Rajiv Kumar, James M. Allan, Christine J. Harrison, Anthony V. Moorman, Ajay Vora, Eve Roman, Sivaramakrishna Rachakonda, Sally E. Kinsey, Eamonn Sheridan, Pamela D. Thompson, Julie A. Irving, Rolf Koehler, Per Hoffmann, Markus M. Nöthen, Stefanie Heilmann-Heimbach, Karl-Heinz Jöckel, Douglas F. Easton, Paul D. P. Pharaoh, Alison M. Dunning, Julian Peto, Frederico Canzian, Anthony Swerdlow, Rosalind A. Eeles, Zsofia Kote-Jarai, Kenneth Muir, Nora Pashayan, The PRACTICAL consortium, Brian E. Henderson, Christopher A. Haiman, Sara Benlloch, Fredrick R. Schumacher, Ali Amin Al Olama, Sonja I. Berndt, David V. Conti, Fredrik Wiklund, Stephen Chanock, Victoria L. Stevens, Catherine M. Tangen, Jyotsna Batra, Judith Clements, Henrik Gronberg, Johanna Schleutker, Demetrius Albanes, Stephanie Weinstein, Alicja Wolk, Catharine West, Lorelei Mucci, Géraldine Cancel-Tassin, Stella Koutros, Karina Dalsgaard Sorensen, Lovise Maehle, David E. Neal, Ruth C. Travis, Robert J. Hamilton, Sue Ann Ingles, Barry Rosenstein, Yong-Jie Lu, Graham G. Giles, Adam S. Kibel, Ana Vega, Manolis Kogevinas, Kathryn L. Penney, Jong Y. Park, Janet L. Stanford, Cezary Cybulski, Børge G. Nordestgaard, Hermann Brenner, Christiane Maier, Jeri Kim, Esther M. John, Manuel R. Teixeira, Susan L. Neuhausen, Kim De Ruyck, Azad Razack, Lisa F. Newcomb, Davor Lessel, Radka Kaneva, Nawaid Usmani, Frank Claessens, Paul A. Townsend, Manuela Gago-Dominguez, Monique J. Roobol, Florence Menegaux, Mel Greaves, Martin Zimmerman, Claus R. Bartram, Martin Schrappe, Martin Stanulla, Kari Hemminki, Richard S. Houlston

**Affiliations:** 1 Division of Genetics and Epidemiology, The Institute of Cancer Research, Sutton, Surrey, SM2 5NG UK; 2 Division of Molecular Genetic Epidemiology, German Cancer Research Centre, 69120 Heidelberg, Germany; 3 Northern Institute for Cancer Research, Newcastle University, Newcastle upon Tyne, NE2 4HH UK; 4 Wolfson Childhood Cancer Research Centre, Northern Institute for Cancer Research, Newcastle University, Newcastle upon Tyne, NE1 7RU UK; 5 Department of Haematology, Great Ormond Street Hospital, London, WC1N 3JH UK; 6 Department of Health Sciences, University of York, York, YO10 5DD UK; 7 Department of Paediatric and Adolescent Haematology and Oncology, Leeds General Infirmary, Leeds, LS1 3EX UK; 8 Medical Genetics Research Group, Leeds Institute of Molecular Medicine, University of Leeds, Leeds, LS9 7TF UK; 9 Paediatric and Familial Cancer Research Group, Institute of Cancer Sciences, St. Mary’s Hospital, Manchester, M13 9WL UK; 10 Department of Human Genetics, Institute of Human Genetics, University of Heidelberg, 69120 Heidelberg, Germany; 11 Department of Genomics, Institute of Human Genetics, Life & Brain Centre, University of Bonn, D-53012 Bonn, Germany; 12 Department of Biomedicine, Human Genomics Research Group, University Hospital and University of Basel, 4031 Basel, Switzerland; 13 Institute for Medical Informatics, Biometry and Epidemiology, University Hospital Essen, University of Duisburg–Essen, 45147 Essen, Germany; 14 Department of Oncology, Centre for Cancer Genetic Epidemiology, University of Cambridge, Cambridge, CB1 8RN UK; 15 Department of Public Health and Primary Care, Centre for Cancer Genetic Epidemiology, University of Cambridge, Cambridge, CB1 8RN UK; 16 Department of Oncology, Centre for Cancer Genetic Epidemiology, University of Cambridge, Strangeways Laboratory, Cambridge, CB1 8RN UK; 17 Department of Non-Communicable Disease Epidemiology, London School of Hygiene and Tropical Medicine, London, WC1E 7HT UK; 18 Genomic Epidemiology Group, German Cancer Research Center (DKFZ), 69120 Heidelberg, Germany; 19 Division of Breast Cancer Research, The Institute of Cancer Research, London, SW7 3RP UK; 20 Royal Marsden NHS Foundation Trust, London, SW3 6JJ UK; 21 Institute of Population Health, University of Manchester, Manchester, M13 9PL UK; 22 Warwick Medical School, University of Warwick, Coventry, CV4 7AL UK; 23 Department of Applied Health Research, University College London, London, WC1E 7HB UK; 24 Centre for Evolution and Cancer, Institute of Cancer Research, Sutton, Surrey, SM2 5NG UK; 25 Department of Paediatric Haematology and Oncology, Hannover Medical School, 30625 Hannover, Germany; 26 General Paediatrics, University Hospital Schleswig-Holstein, 24105 Kiel, Germany; 27 Center for Primary Health Care Research, Lund University, 221 00 Lund, Sweden; 28 Department of Preventive Medicine, Keck School of Medicine, University of Southern California/Norris Comprehensive Cancer Center, Los Angeles, CA 90033 USA; 29 Department of Public Health and Primary Care, Centre for Cancer Genetic Epidemiology, Strangeways Research Laboratory, University of Cambridge, Cambridge, CB2 0SP UK; 30 The Institute of Cancer Research, London, SM2 5NG UK; 31 Department of Epidemiology and Biostatistics, Case Western Reserve University, Cleveland, OH 44106 USA; 32 Seidman Cancer Center, University Hospitals, Cleveland, OH 44106 USA; 33 Department of Clinical Neurosciences, University of Cambridge, Cambridge, CB2 2PY UK; 34 Division of Cancer Epidemiology and Genetics, National Cancer Institute, NIH, Bethesda, MD 20814 USA; 35 Department of Medical Epidemiology and Biostatistics, Karolinska Institute, Stockholm, 171 77 Sweden; 36 Epidemiology Research Program, American Cancer Society, 250 Williams Street, Atlanta, GA 30303 USA; 37 SWOG Statistical Center, Fred Hutchinson Cancer Research Center, Seattle, WA 98109-1024 USA; 38 Australian Prostrate Cancer BioResource (APCB), Australian Prostate Cancer Research Centre-Qld, Institute of Health and Biomedical Innovation and School of Biomedical Science, Queensland University of Technology, Brisbane, 4001 QLD Australia; 39 Translational Research Institute, Brisbane, 4102 QLD Australia; 40 Department of Medical Biochemistry and Genetics, Institute of Biomedicine, University of Turku, Turku, FI-20014 Finland; 41 Tyks Microbiology and Genetics, Department of Medical Genetics, Turku University Hospital, Turku, 20521 Finland; 42 BioMediTech, University of Tampere, Tampere, 33100 Finland; 43 Division of Nutritional Epidemiology, Institute of Environmental Medicine, Karolinska Institutet, Solna, SE-171 77 Sweden; 44 Institute of Cancer Sciences, University of Manchester, Manchester Academic Health Science Centre, Radiotherapy Related Research, The Christie Hospital NHS Foundation Trust, Manchester, M13 9PL UK; 45 Department of Epidemiology, Harvard School of Public Health, Boston, MA 02115 USA; 46 CeRePP, Pitie-Salpetriere Hospital, Paris, 75020 France; 47 UPMC Univ Paris 06, GRC N°5 ONCOTYPE-URO, CeRePP, Tenon Hospital, Paris, 75020 France; 48 Department of Molecular Medicine, Aarhus University Hospital, Aarhus, DK-8200 Denmark; 49 Department of Clinical Medicine, Aarhus University, Aarhus, 8000 Denmark; 50 Department of Medical Genetics, Oslo University Hospital, Oslo, 0424 Norway; 51 Department of Oncology, Addenbrooke’s Hospital, University of Cambridge, Cambridge, CB2 0QQ UK; 52 Li Ka Shing Centre, Cancer Research UK Cambridge Research Institute, Cambridge, CB2 0RE UK; 53 Cancer Epidemiology, Nuffield Department of Population Health, University of Oxford, Oxford, OX3 7LF UK; 54 Department of Surgical Oncology, Princess Margaret Cancer Centre, Toronto, M5G 2C4 Canada; 55 Department of Radiation Oncology, Icahn School of Medicine at Mount Sinai, New York, NY 10029-5674 USA; 56 Department of Genetics and Genomic Sciences, Icahn School of Medicine at Mount Sinai, New York, NY 10029-5674 USA; 57 Centre for Molecular Oncology, Barts Cancer Institute, John Vane Science Centre, Queen Mary University of London, London, EC1M 6BQ UK; 58 Cancer Epidemiology Centre, The Cancer Council Victoria, Melbourne, 3004 VIC Australia; 59 Centre for Epidemiology and Biostatistics, Melbourne School of Population and Global Health, The University of Melbourne, Melbourne, 3010 Australia; 60 Division of Urologic Surgery, Brigham and Womens Hospital, Boston, MA 02115 USA; 61 Fundación Pública Galega de Medicina Xenómica-SERGAS, Grupo de Medicina Xenómica, CIBERER, IDIS, Santiago de Compostela, 15706 Spain; 62 Centre for Research in Environmental Epidemiology (CREAL), Barcelona Institute for Global Health (ISGlobal), Barcelona, 08036 Spain; 63 CIBER Epidemiología y Salud Pública (CIBERESP), Madrid, 28029 Spain; 64 IMIM (Hospital del Mar Research Institute), Barcelona, 08003 Spain; 65 Universitat Pompeu Fabra (UPF), Barcelona, 08005 Spain; 66 Channing Division of Network Medicine, Department of Medicine, Brigham and Women’s Hospital/Harvard Medical School, Boston, MA 02115 USA; 67 Department of Cancer Epidemiology, Moffitt Cancer Center, Tampa, 33612 USA; 68 Division of Public Health Sciences, Fred Hutchinson Cancer Research Center, Seattle, WA 98109 USA; 69 Department of Epidemiology, School of Public Health, University of Washington, Seattle, WA 98195 USA; 70 International Hereditary Cancer Center, Department of Genetics and Pathology, Pomeranian Medical University, Szczecin, 70-204 Poland; 71 Faculty of Health and Medical Sciences, University of Copenhagen, Copenhagen, DK-2200 Denmark; 72 Department of Clinical Biochemistry, Herlev and Gentofte Hospital, Copenhagen University Hospital, Herlev, 2730 Denmark; 73 Division of Clinical Epidemiology and Aging Research, German Cancer Research Center (DKFZ), Heidelberg, 69120 Germany; 74 German Cancer Consortium (DKTK), German Cancer Research Center (DKFZ), Heidelberg, 69120 Germany; 75 Division of Preventive Oncology, German Cancer Research Center (DKFZ) and National Center for Tumor Diseases (NCT), Heidelberg, 69120 Germany; 76 Institute for Human Genetics, University Hospital Ulm, Ulm, 89081 Germany; 77 Department of Genitourinary Medical Oncology, The University of Texas MD Anderson Cancer Center, Houston, TX 77030 USA; 78 Cancer Prevention Institute of California, Fremont, CA 94538 USA; 79 Department of Health Research & Policy (Epidemiology) and Stanford Cancer Institute, Stanford University School of Medicine, Stanford, CA 94305-5456 USA; 80 Department of Genetics, Portuguese Oncology Institute of Porto, Porto, 4200-072 Portugal; 81 Biomedical Sciences Institute (ICBAS), University of Porto, Porto, 4050-313 Portugal; 82 Department of Population Sciences, Beckman Research Institute of the City of Hope, Duarte, CA 91010 USA; 83 Faculty of Medicine and Health Sciences, Basic Medical Sciences, Ghent University, Gent, 9000 Belgium; 84 Department of Surgery, Faculty of Medicine, University of Malaya, Kuala Lumpur, 50603 Malaysia; 85 Department of Urology, University of Washington, Seattle, WA 98195 USA; 86 Institute of Human Genetics, University Medical Center Hamburg-Eppendorf, Hamburg, 20246 Germany; 87 Molecular Medicine Center, Department of Medical Chemistry and Biochemistry, Medical University, Sofia, 1431 Bulgaria; 88 Department of Oncology, Cross Cancer Institute, University of Alberta, Edmonton, T6G 1Z2 AB Canada; 89 Division of Radiation Oncology, Cross Cancer Institute, Edmonton, T6G 2R7 AB Canada; 90 Department of Cellular and Molecular Medicine, Molecular Endocrinology Laboratory, KU Leuven, Leuven, 3000 Belgium; 91 Institute of Cancer Sciences, Manchester Cancer Research Centre, University of Manchester, Manchester Academic Health Science Centre, St. Mary’s Hospital, Manchester, M13 9PL UK; 92 Genomic Medicine Group, Galician Foundation of Genomic Medicine, Instituto de Investigacion Sanitaria de Santiago de Compostela (IDIS), Complejo Hospitalario Universitario de Santiago, Servicio Galego de Saúde, SERGAS, Santiago de Compostela, 15706 Spain; 93 Moores Cancer Center, University of California San Diego, La Jolla, CA 92093 USA; 94 Department of Urology, Erasmus University Medical Center, Rotterdam, 3015 The Netherlands; 95 Cancer & Environment Group, Center for Research in Epidemiology and Population Health (CESP), INSERM, University Paris-Sud, University Paris-Saclay, Villejuif, 94800 France

Correction to: *Nature Communications*; 10.1038/s41467-018-03178-z; published online 9 April 2018

The original version of this Article contained an error in the spelling of a member of the PRACTICAL Consortium, Manuela Gago-Dominguez, which was incorrectly given as Manuela Gago Dominguez. This has now been corrected in both the PDF and HTML versions of the Article. Furthermore, in the original HTML version of this Article, the order of authors within the author list was incorrect. The PRACTICAL consortium was incorrectly listed after Richard S. Houlston and should have been listed after Nora Pashayan. This error has been corrected in the HTML version of the Article; the PDF version was correct at the time of publication.

