# Supplementary material for: Genome-wide association study identifies susceptibility loci for B-cell childhood acute lymphoblastic leukemia
Source: Nat Commun. 2018 Apr 9;9:1340. doi: 10.1038/s41467-018-03178-z (PMC5890276; doi:10.1038/s41467-018-03178-z)
Supplement: Supplementary file 1 — Supplementary Information [file 41467_2018_3178_MOESM1_ESM.pdf]

SUPPLEMENTARY FIGURES

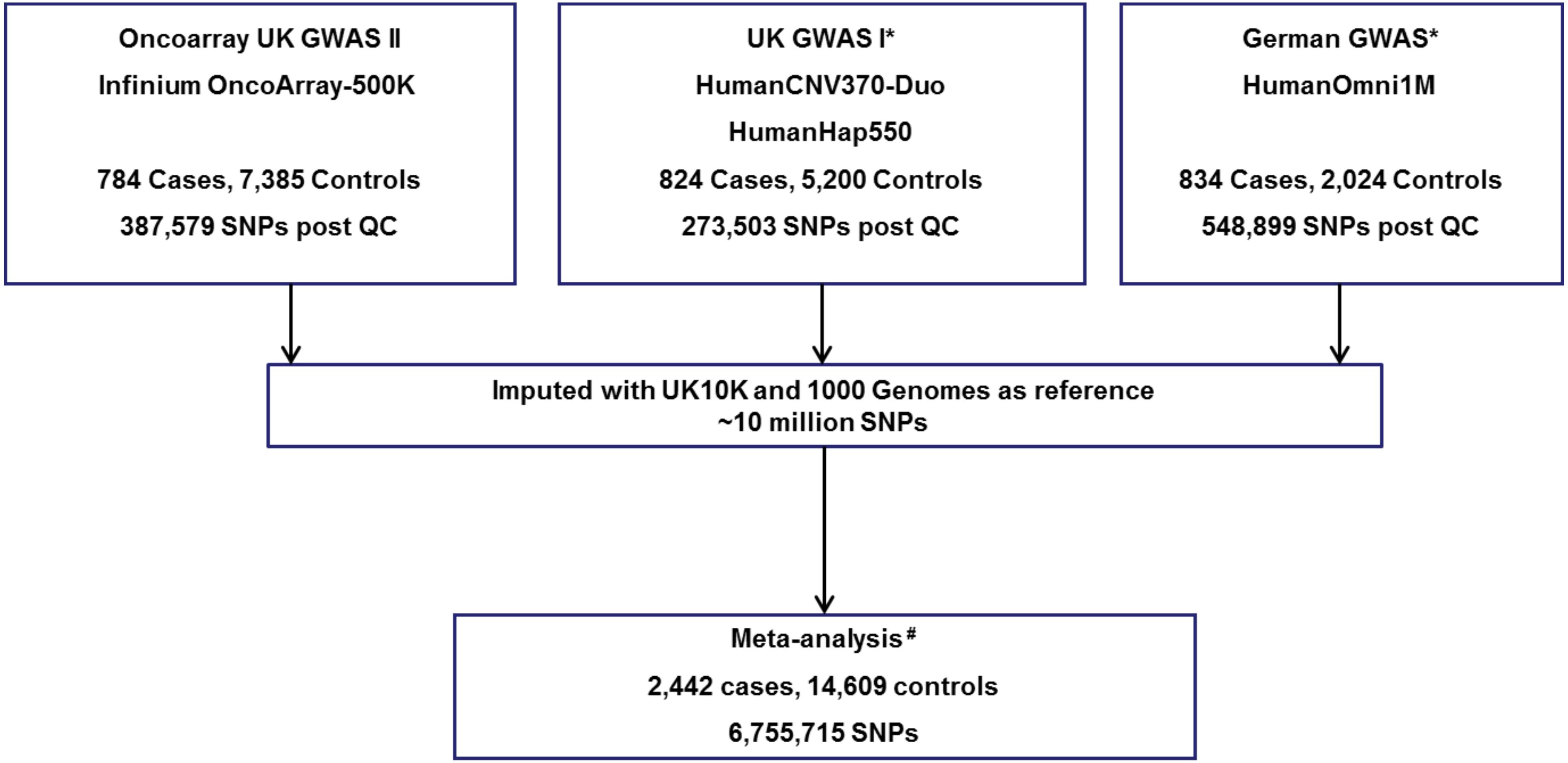

Supplementary Figure 1: Overall study scheme. * Details of UK GWAS I and German GWAs are previously described [1-3]. #Post imputation QC criteria included: MAF>0.01; INFO score>0.8 and HWE *P*-value>$1x10^{-5}$.

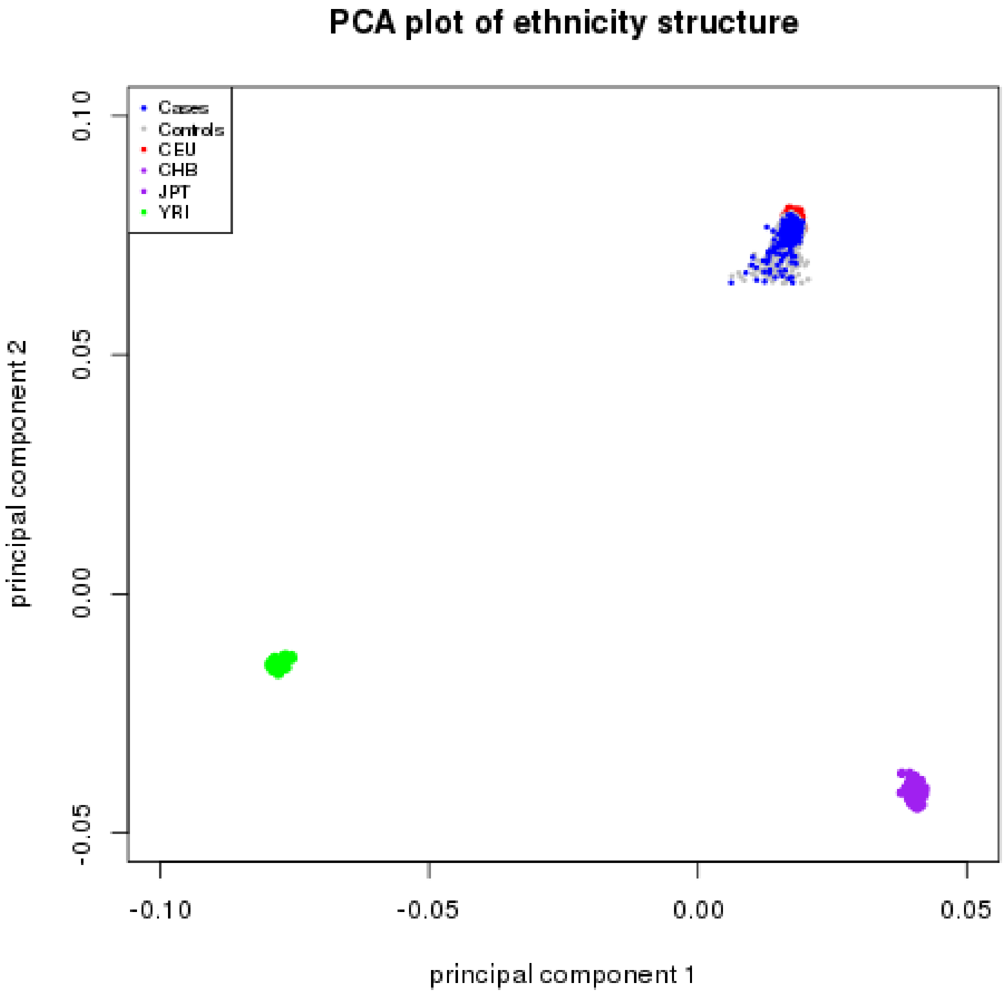

Supplementary Figure 2: Principal component analysis plot of ethnicity structure in UK GWAS II post sample QC

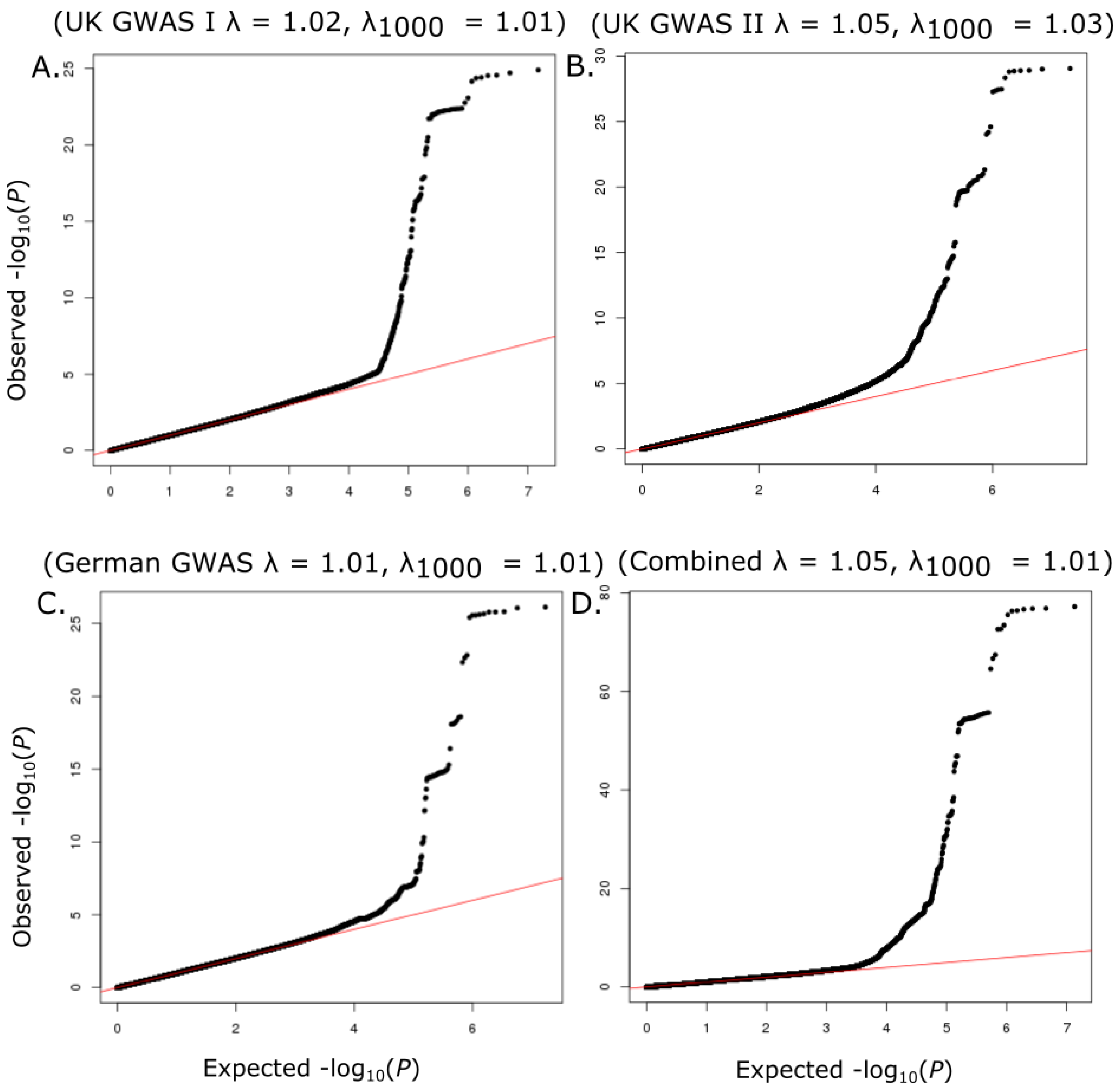

Supplementary Figure 3: QQ plot post imputation and genomic corrections for (A) UK GWAS I, (B) UK GWAS II and (C) German GWAS (D) Meta-analysis showing combined λ and $λ_{1000}$.

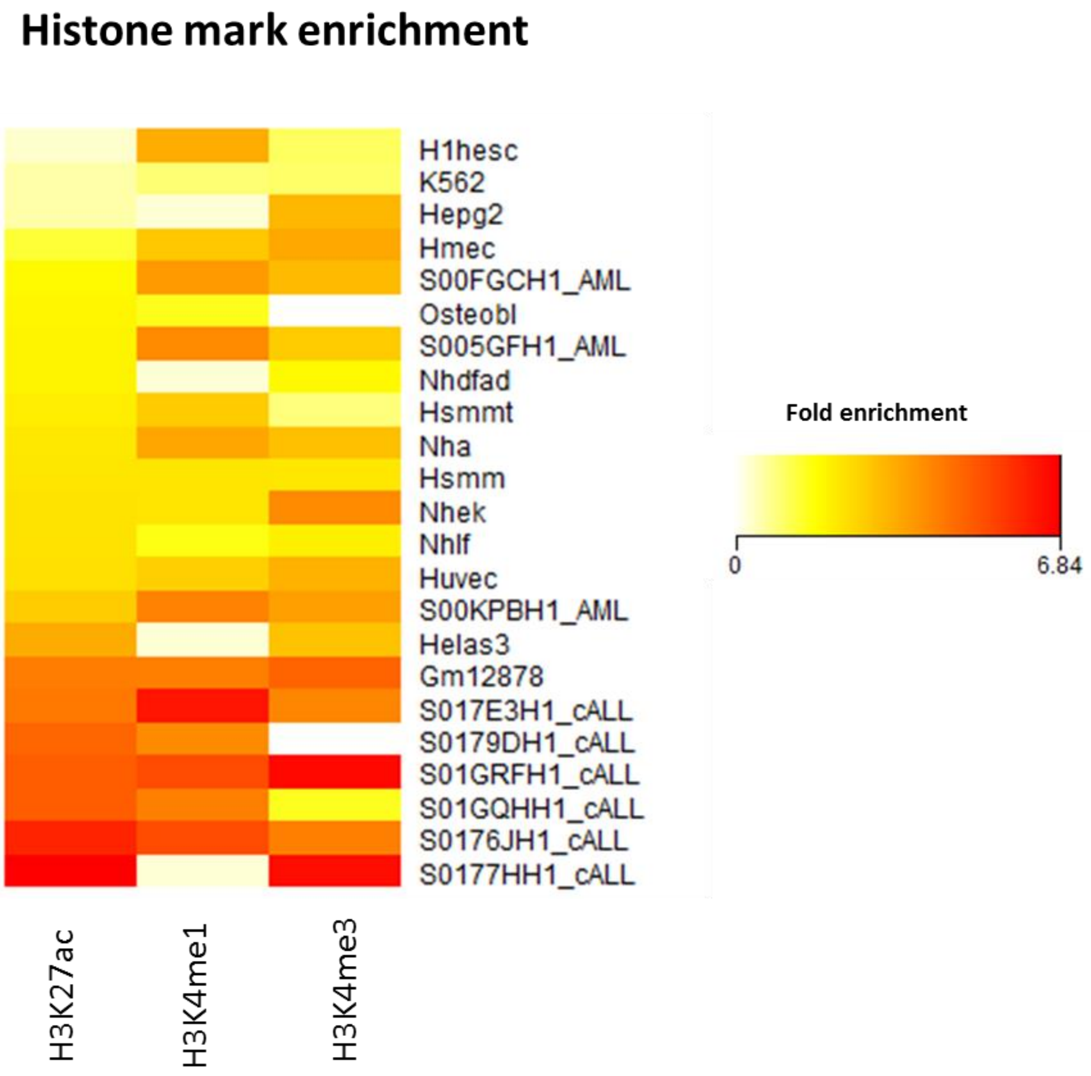

Supplementary Figure 4: Tissue specific histone mark enrichment. The heatmap shows enrichment scores for histone marks of active chromatin H3K27ac, H3K4me1 and H3K4me3, using ChIP-Seq data from 14 cell-types from ENCODE (www.encodeproject.org), three AML and six childhood ALL cell lines obtained from the Blue-print Epigenome data base. White colouring means no data was available. H1-hesc: embryonic stem cells; K562: leukaemia; Hepg2: hepatocellular carcinoma; Hmec: epithelial cells; Osteobl: osteoblasts; Nhdfad: fibrobalsts; Hsmm: myeoblasts; Nhek: Normal Human Epidermal Keratinocytes; Nhlf: lung fibroblasts; Huvec: human umbilical vein endothelial cell; Helas3: cervical adenocarcinoma; Gm12878: lymphoblastoid cell line; *_AML: acute myeloid leukaemia, *_cALL: childhood acute lymphoblastic leukaemia cells.

A. 10q26.13; ILMN_1704571 (FAM53B); Whole blood Locus plot

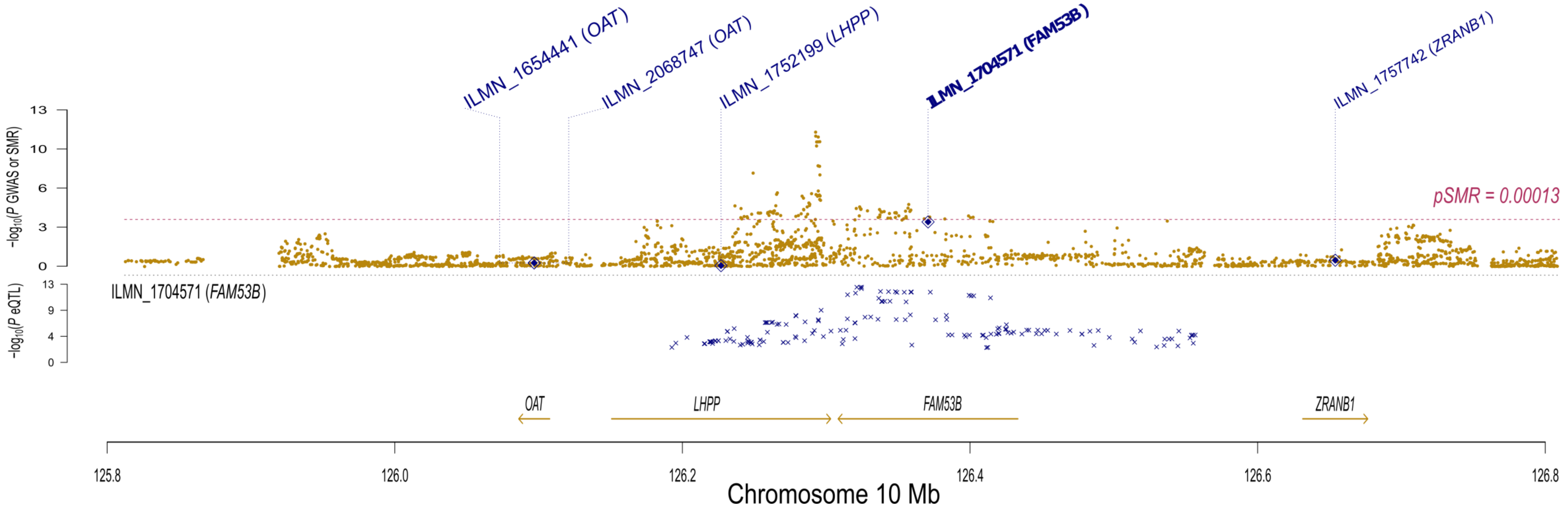

B. 10q26.13; ILMN_1704571 (*FAM53B*); Whole blood effect size plot

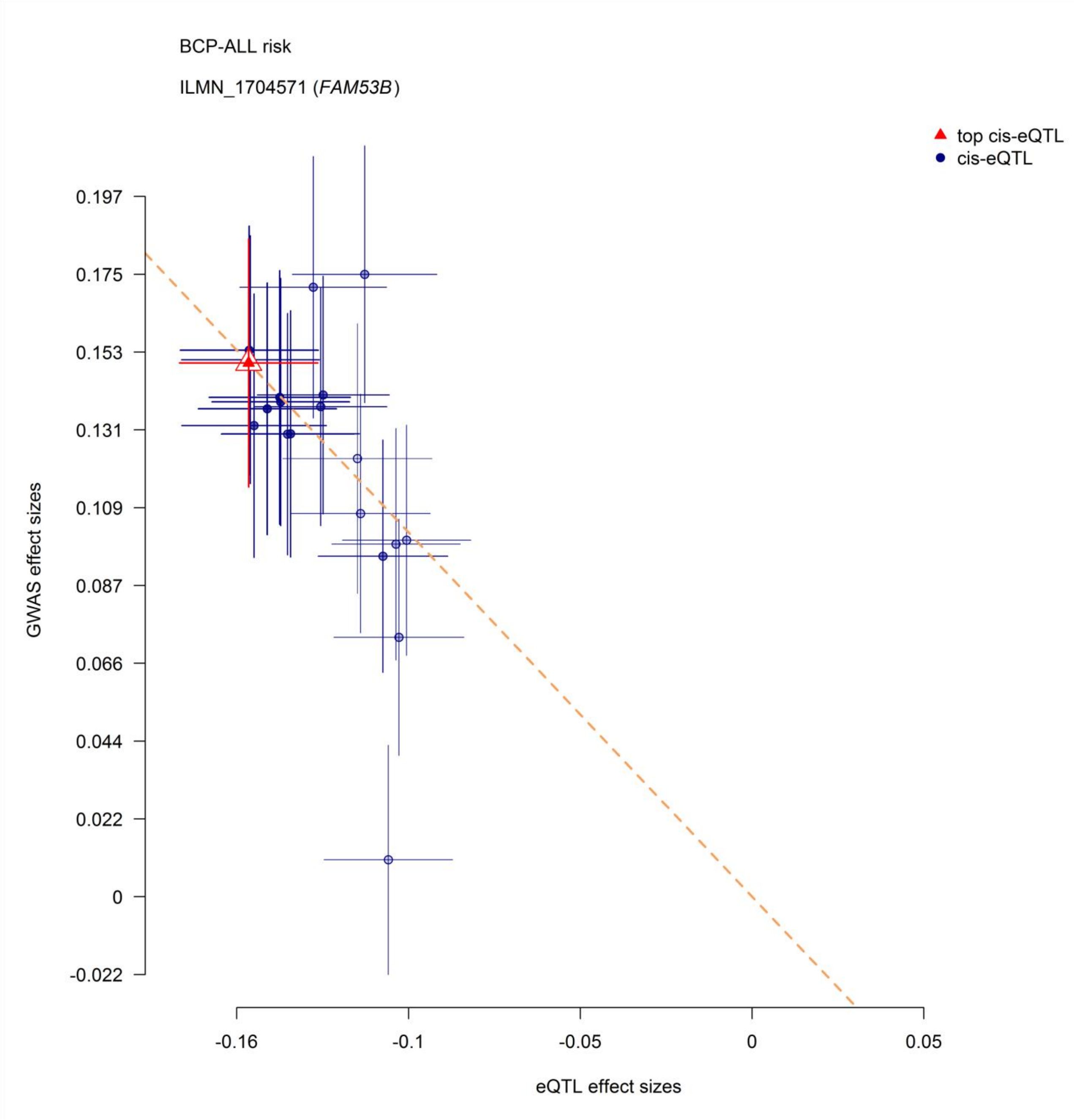

C. 10p12.2; ILMN_2152465 (*PIP4K2A*); Whole blood Locus plot

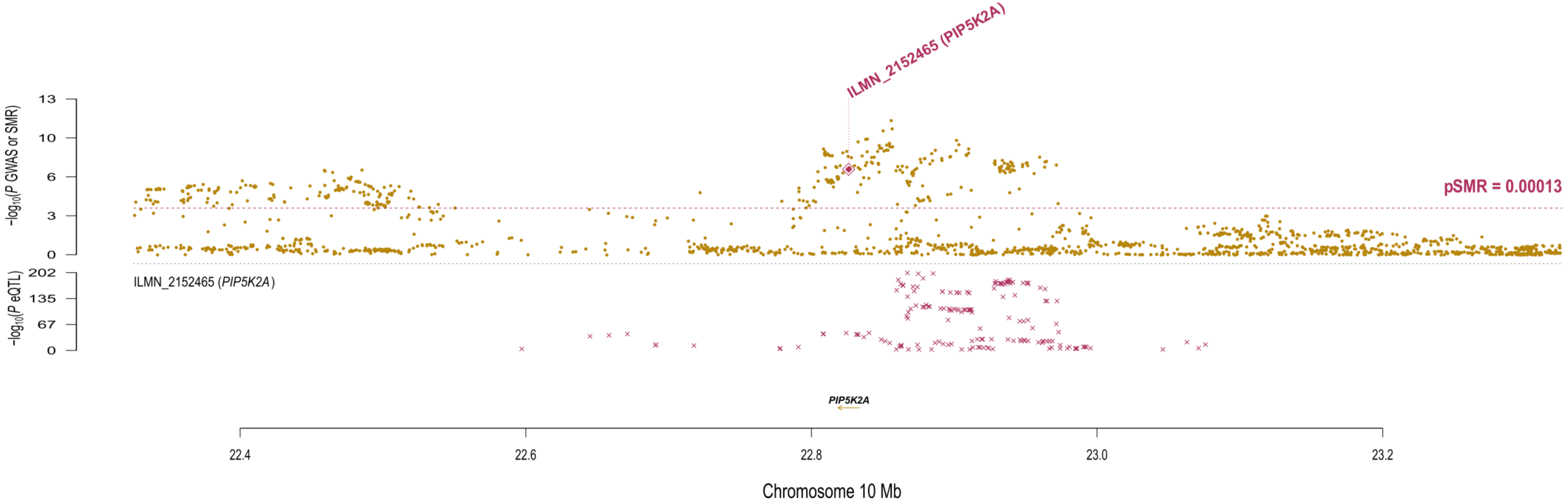

D. 10p12.2; ILMN_2152465 (*PIP4K2A*); Whole blood Locus plot

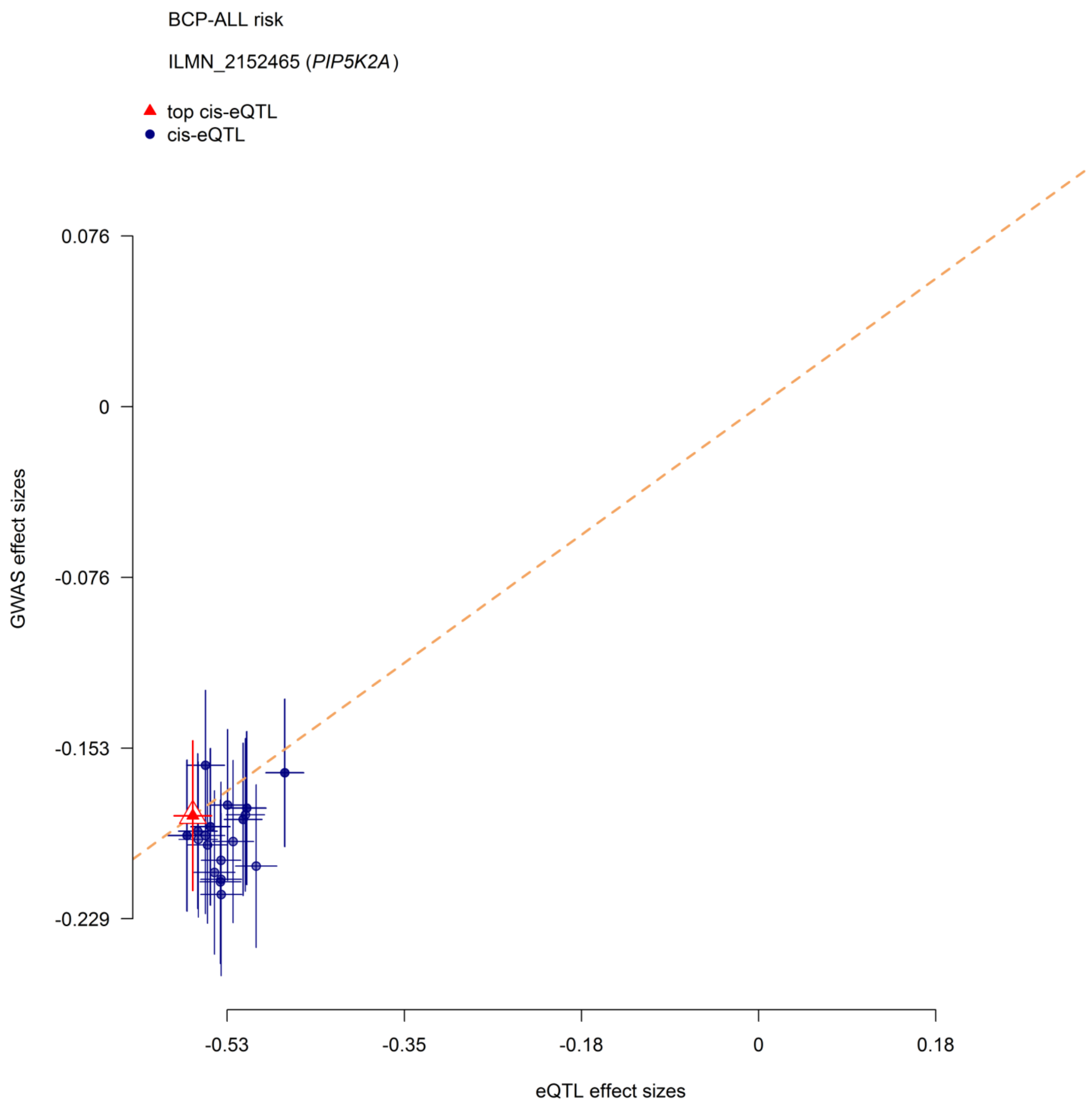

Supplementary Figure 5: SMR eQTL plots at loci: 10q26.13 (A,B) and 10p12.2(C,D). At each locus: (Locus plots: A,C) Upper panel- Brown dots represent *P*-values for GWAS SNPs, diamonds represent *P*-values for probes from the SMR test, (ii) lower panel – crosses show eQTL *P*-values of SNPs from whole blood, genes passing the SMR (*i.e.* $P_{SMR} < 1.3x10^{-4}$) and HEIDI (*i.e.* $P_{HEIDI} > 0.05$) tests are highlighted in red, the top and bottom plots include all SNPs mapping to the region in the GWAS and eQTL summary data, rather than only SNPs common to both datasets. (Effect plots: B,D) Effect sizes of SNPs (used in the HEIDI test) from GWAS meta-analysis plotted against those for SNPs from whole blood eQTL data. The orange dashed lines correspond to the estimate of b_{xy}, at the top *cis*-eQTL. Error bars correspond to standard errors of SNP effects. Note: SMR calculates eQTL *P*-values from beta and standard error values, therefore reported eQTL *P*-values may not coincide with exactly with those reported by Westra *et al[4]*.

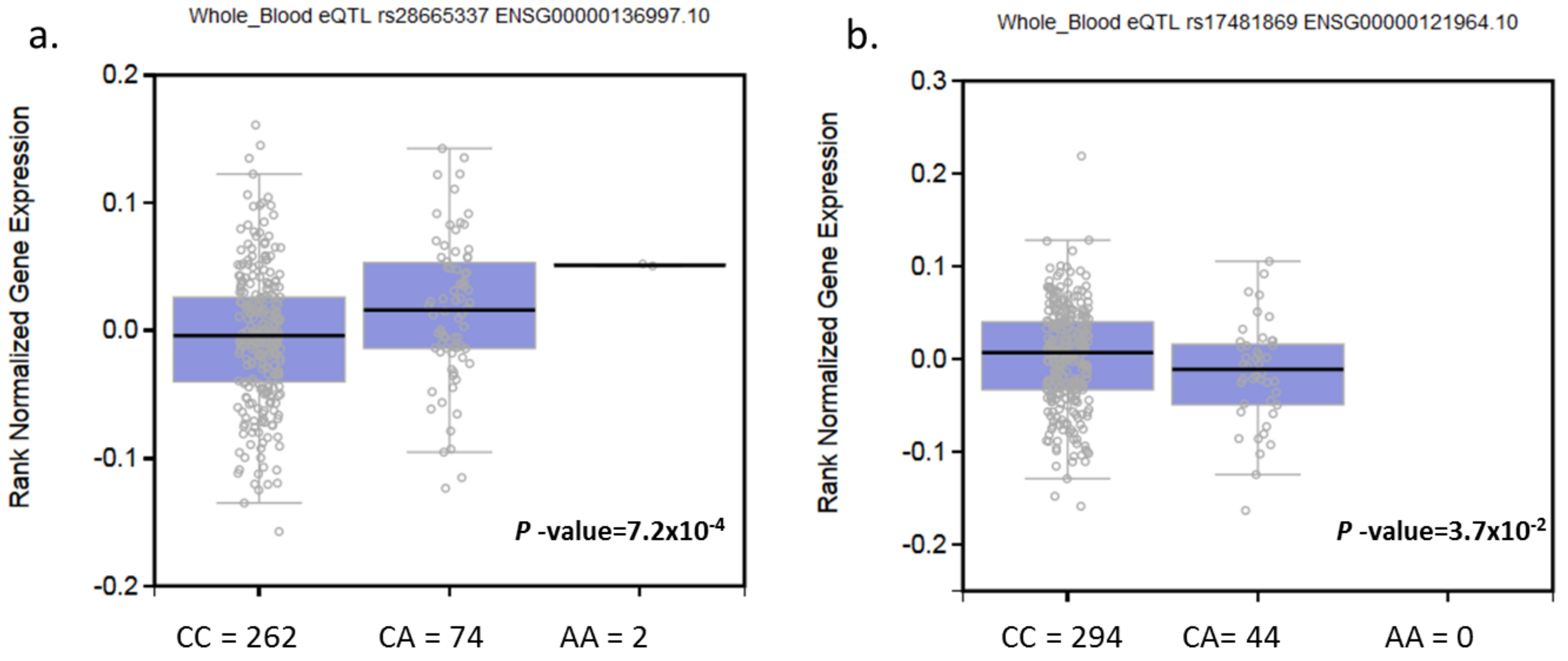

Supplementary Figure 6 (a) eQTL analysis of rs28665337 genotypes and expression of *MYC* (b) rs17481869 genotypes and expression of *GTDC1*. Plots were generated using GTEX Analysis Release V6p. X-axes are the number of alleles of the two SNPs belonging to each genotype group. Y-axis shows gene expression obtained from RNA-seq and rank normalisation was performed to normalise expression data. The box plot displays ranked normalised gene expression in median, 1st and 3rd quartiles, 1.5 interquartile range of 1st and 3rd quartiles. The *P*-values are generated from a T-test statistic. The T-statsitics are as follows, (i) for rs28665337: 3.1 (standard error (se): 0.059); (ii) for rs17481869: -2.1 (se:0.044).

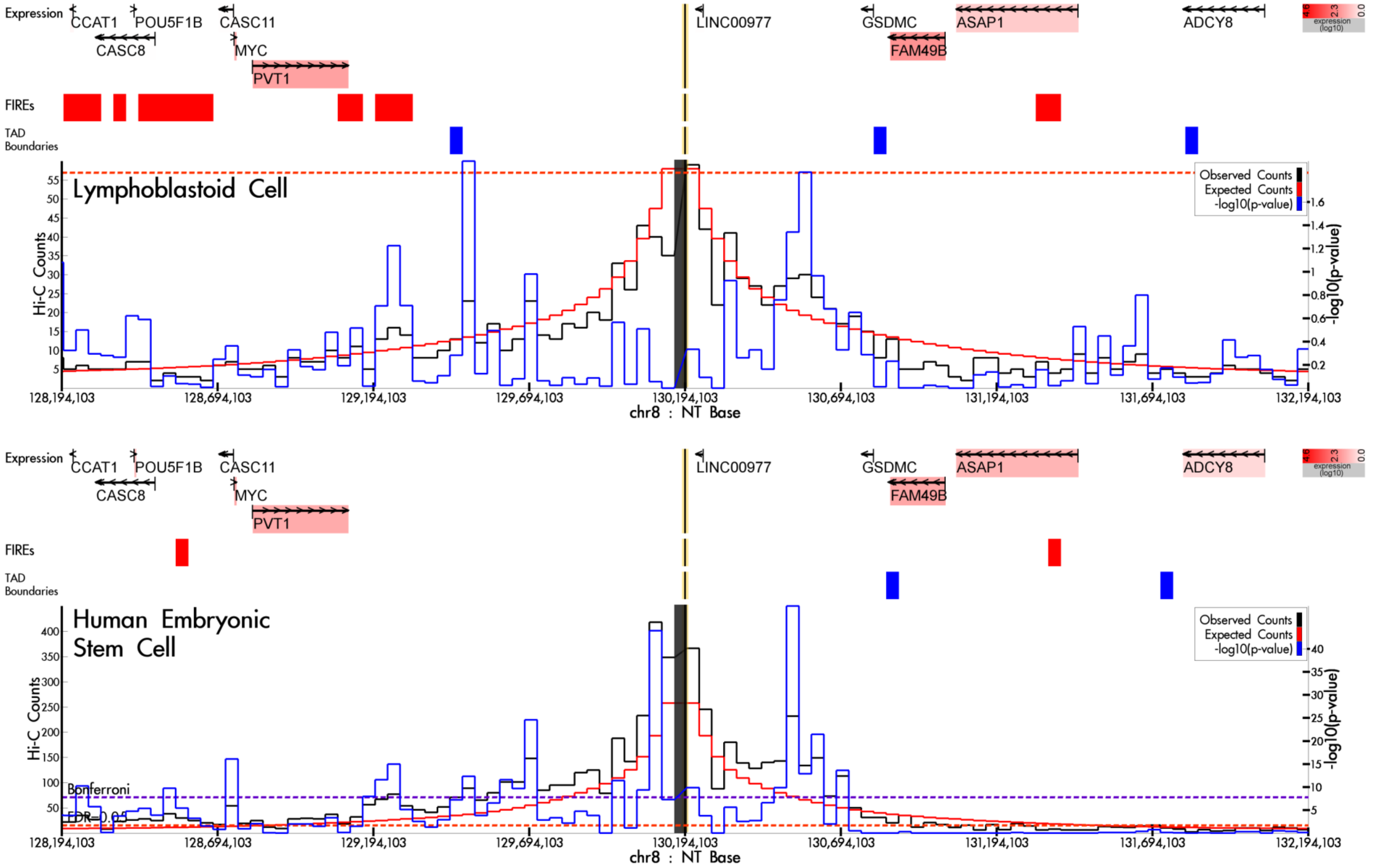

Supplementary Figure 7: rs28665337 (8q24.21) showing a significant long range chromatin interaction with the promoter region of the gene *MYC* (*P*-value<$1x10^{-16}$) in H1 embryonic stem cells. Virtual 4C plot was generated using the HUGIN browser[5]. Graded red colouring of gene names indicates significant gene expression. Left Y-axis shows Hi-C counts while right Y-axis shows $-\log_{10}$ *P*-values. Vertical dark line indicates anchor positions of variant rs28665337.FIRE: Frequently Interacting Regions; TAD: Topologically Associating Domain. *P*-values generated using 'Fit-Hi-C'[6]

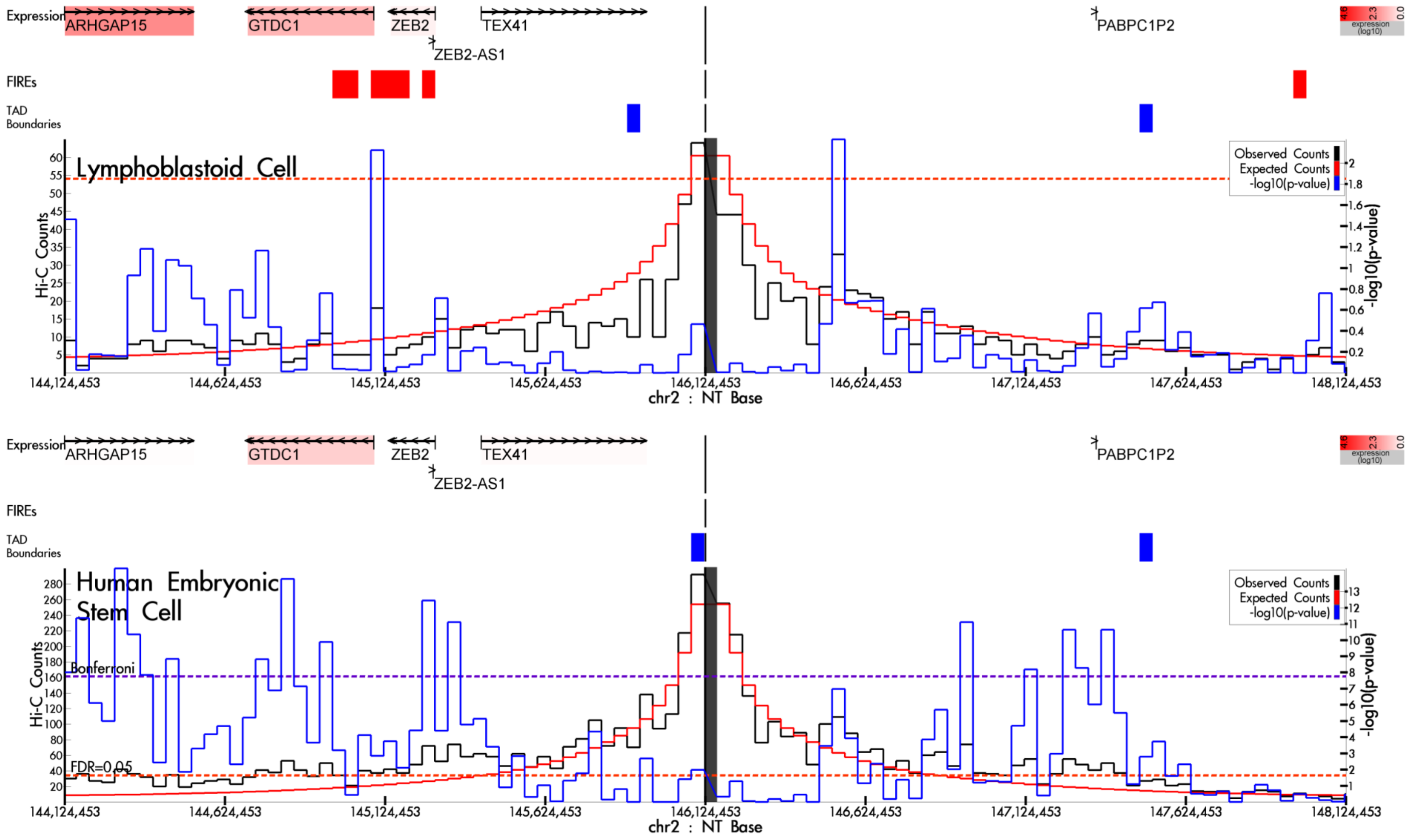

Supplementary Figure 8: rs17481869 (2q22.3) showing a significant long range chromatin interaction with the promoter region of the gene *GTDC1* (*P*-value<1x10^{-2}) in Lymphoblastoid cell lines. Virtual 4C plot was generated using the HUGIN browser[5]. Graded red colouring of gene names indicates significant gene expression. Left Y-axis shows Hi-C counts while right Y-axis shows –$\log_{10}$ *P*-values. Vertical dark line indicates anchor positions of variant rs17481869. FIRE: Frequently Interacting Regions; TAD: Topologically Associating Domain. *P*-values are calculated based on observed counts vs expected counts. . *P*-values generated using 'Fit-Hi-C'[6].

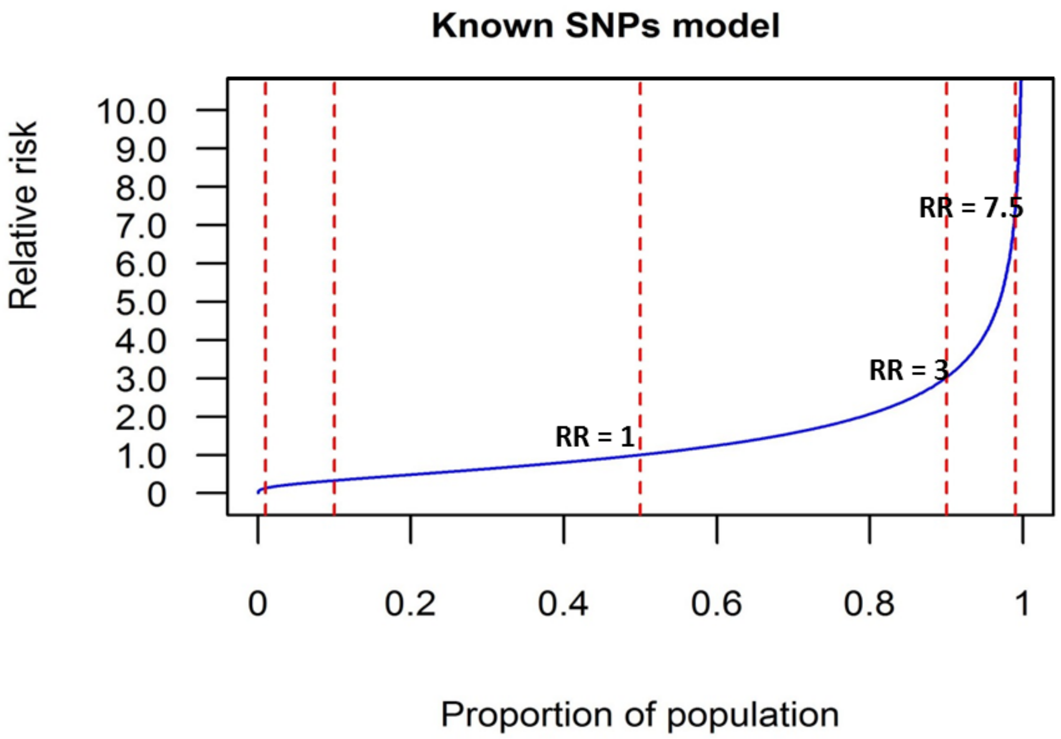

Supplementary Figure 9: Population distribution of BCP-ALL relative risk scores ordered by genetic risk (risk is relative to population median risk). The blue line plots the distribution of relative risk (RR) across the population; the red lines correspond to 1st, 10th, 50th, 90th and 99th centiles. The RR figures presented in black are the average in the highest (i) 10th and (ii) 1 centile of genetic risk.

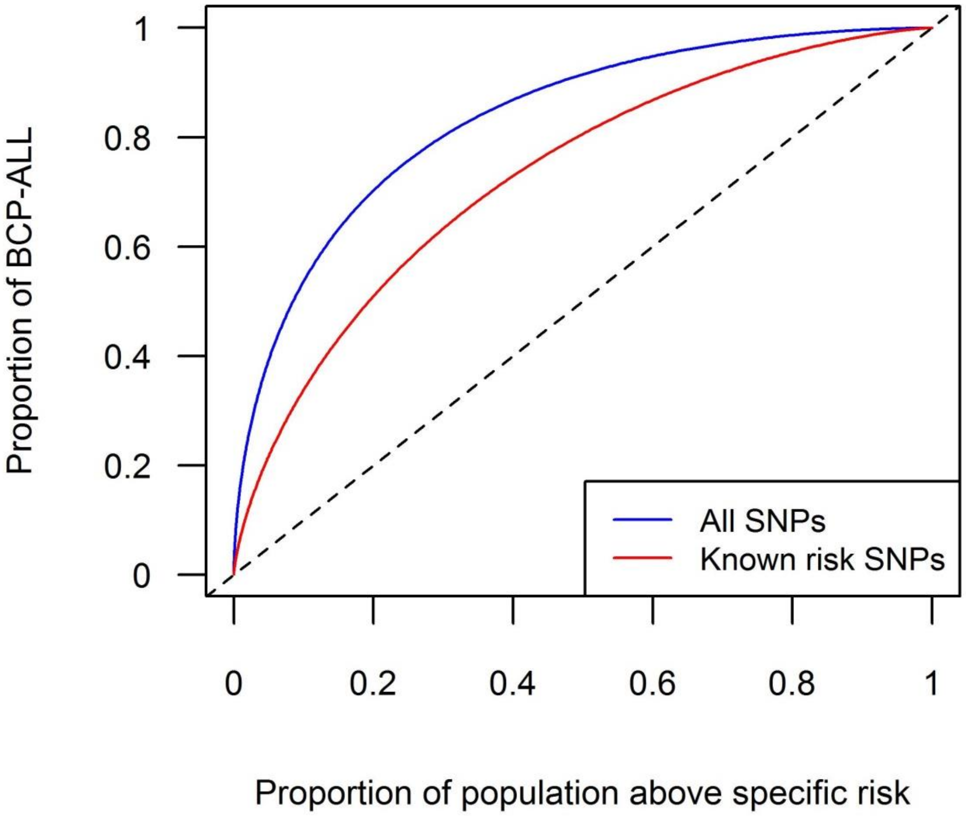

Supplementary Figure 10: ROC curve for BCP-ALL SNPs. The blue line represents all the BCP-ALL predisposition factors including environmental and genetic; the red line represents the polygenic risk score (PRS) based on the 11 genetic variants. Area under the curve for known SNPs was 0.73 under model assuming a sibling risk of 3.2.

SUPPLEMENTARY TABLES

Supplementary Table 1: Details of the quality control filters applied to Oncoarray UK GWAS II. (a) Samples were excluded due to call rate (< 95% or failed genotyping), ethnicity (principle components analysis or other samples reported to be not of white, European descent), relatedness (any individuals found to be duplicated or related within or between data sets through identity by state) or sex discrepancy. (b) Genotyped single nucleotide polymorphisms (SNPs) with a call rate < 95% were excluded as were those with a minor allele frequency (MAF) < 0.01 or displaying significant deviation from Hardy-Weinberg equilibrium (HWE) (i.e. $P < 1x10^{-5}$). Imputed SNPs with information score < 0.8 and MAF < 0.01 were excluded.

**a. Sample QC**	**UK GWAS II**	
	Cases	Controls
Pre-quality control	1,021	7,519
Sex discrepancy	13	0
Call rate fail/ Heterozygosity rate (<95%)	88	35
Related Individuals	13	67
Non-European Ancestry	219	56
Post-quality control†	784	7,385

**b. SNP QC**	**UK GWAS II**
Genotyping Platform	Infinium OncoArray-500K BeadChip
Pre-quality control	473,991
Call rate fail (<95%)	8
HWE fail (P-value<$1x10^{-5}$)	20
MAF (< 0.01)	75,937
Differential case-control genotype rate	10448
Post-quality control†	387,579

† Filters for QC were performed simultaneously so numbers for each criterion may not sum to total removed.

Supplementary Table 2: *P*-values of sentinel SNPs from previously published GWAS together with their new *P*-values from the current meta-analyses. Also shown are the new top hits from each loci and their r^2 and D' with the previous SNPs. (a) Previously published top SNPs at risk loci taken from Papaemmanuil *et al.*, Migliorini *et al.* and Vijayakrishnan *et al*. [1-3, 7]. (b) *P*-values from the current meta-analysis. (c) SNPs that map to previously published risk loci that have a more significant *P*-value in the current meta-analyses. (d) rs4762284 achieves genome wide significance when combined with a German replication series as shown in Vijayakrishnan *et al*[3]. *P*-values are calculated from pooling the beta values and standard errors assuming a fixed model for each SNP from each study.

Chr	Locus (gene)	Previous top SNP[a]	Meta-analysis *P*-value[b]	Top SNP from Meta-analysis[c]	Position	r^2	D'	Odds ratio (95%CI)	*P*-value
7	7p12.2 (*IKZF1*)[2]	rs4132601	$6.33x10^{-55}$	rs10230978	50477144	0.97	0.99	1.75 (1.63-1.88)	1.89×10^{-56}
9	9p21.3 (*CDKN2A*)[3]	rs3731249	$1.09x10^{-27}$	-	21970916	-	-	2.65 (2.22-3.17)	1.09×10^{-27}
9	9p21.3 (*CDKN2A*)[5]	rs3731217	$7.24x10^{-14}$	-	21984661	-	-	0.70 (0.64-0.77)	7.24×10^{-14}
10	10q21.2(*ARID5B*)[1]	rs7089424	$1.98x10^{-73}$	rs10821936	63723577	-	-	0.53 (0.50-0.57)	5.77×10^{-78}
10	10p14 (*GATA3*)[1]	rs3824662	$1.67x10^{-10}$	rs3781093	8101927	0.86	0.97	1.34 (1.22-1.47)	1.18×10^{-10}
10	10p12.2 (*PIP4K2A*)[1]	rs10828317	$2.07x10^{-10}$	rs11013051	22856279	0.85	0.93	0.79 (0.73-0.84)	5.92×10^{-12}
10	10q26.13 (*LHPP*)[4]	rs35837782	$9.35x10^{-11}$	rs12779301	126292655	0.86	0.97	1.26 (1.18-1.35)	6.63×10^{-12}
12	12q23.1 (*ELK3*)[4]	rs4762284[d]	$3.15x10^{-07}$	rs1030137	96616051	0.86	0.96	1.21 (1.12-1.30)	1.35×10^{-07}
14	14q11.2 (*CEBPE*)[2]	rs2239633	$5.39x10^{-14}$	rs2239630	23589349	0.74	0.98	0.73 (0.69-0.78)	2.45×10^{-21}

1. Migliorini, G., et al., *Variation at 10p12.2 and 10p14 influences risk of childhood B-cell acute lymphoblastic leukemia and phenotype.* Blood, 2013. **122**(19): p. 3298-307.
2. Papaemmanuil, E., et al., *Loci on 7p12.2, 10q21.2 and 14q11.2 are associated with risk of childhood acute lymphoblastic leukemia.* Nat Genet, 2009. **41**(9): p. 1006-10.
3. Vijayakrishnan, J., et al., *The 9p21.3 risk of childhood acute lymphoblastic leukaemia is explained by a rare high-impact variant in CDKN2A.* Sci Rep, 2015. **5**: p. 15065.
4. Vijayakrishnan, J., et al., *A genome-wide association study identifies risk loci for childhood acute lymphoblastic leukemia at 10q26.13 and 12q23.1.* Leukemia, 2017. **31**(3): p. 573-579.
5. Sherborne, A.L., et al., *Variation in CDKN2A at 9p21.3 influences childhood acute lymphoblastic leukemia risk.* Nat Genet, 2010. **42**(6): p. 492-4.

Supplementary Table 3: Genotype counts for newly discovered BCP-ALL risk loci and ETV6/RUNX1 subtype. CHR: Chromosome, POS: Position in Hg19 build, A/B: Alleles, *P*-values are calculated from the beta values and standard errors assuming from each study using the frequentist additive model in SNPTEST . rs28665337 and rs7449087 genotypes were imputed in all three cohorts (INFO score >0.97); rs17481869 typed in German GWAS and UK GWAS II.

**UK GWAS 1**			**Alleles**		**Cases**				**Controls**				
**SNP**	**CHR**	**POS**	**A**	**B**	**AA**	**AB**	**BB**	**RAF**	**AA**	**AB**	**BB**	**RAF**	***P*-value**
rs28665337	8	130194104	C	A	600	209	15	0.15	4049	1086	64	0.12	$7.92x10^{-04}$
rs17481869*	2	146124454	C	A	99	26	1	0.11	4503	675	22	0.07	$8.52x10^{-03}$
rs7449087	5	107928071	C	T	474	295	55	0.25	2626	2162	412	0.29	$4.66x10^{-4}$

**German GWAS**					**Cases**				**Controls**				
**SNP**	**CHR**	**POS**	**A**	**B**	**AA**	**AB**	**BB**	**RAF**	**AA**	**AB**	**BB**	**RAF**	***P*-value**
rs28665337	8	130194104	C	A	600	207	27	0.16	1552	443	29	0.12	$7.64x10^{-03}$
rs17481869*	2	146124454	C	A	48	15	0	0.12	1717	295	12	0.08	$1.14\ x10^{-01}$
rs7449087	5	107928071	C	T	451	328	55	0.26	1017	819	188	0.29	$1.12x10^{-2}$

**UK GWAS II**					**Cases**				**Controls**				
**SNP**	**CHR**	**POS**	**A**	**B**	**AA**	**AB**	**BB**	**RAF**	**AA**	**AB**	**BB**	**RAF**	***P*-value**
rs28665337	8	130194104	C	A	561	206	17	0.15	5764	1504	117	0.12	$4.16x10^{-05}$
rs17481869*	2	146124454	C	A	165	51	4	0.13	6338	1002	45	0.07	$2.90\ x10^{-06}$
rs7449087	5	107928071	C	T	448	298	38	0.24	3798	3006	581	0.28	$2.33x10^{-4}$

Supplementary Table 4: Imputation quality scores and concordance between directly sequenced and imputed genotype for SNPs which were genome-wide significant after replication. AA, homozygote for major allele; AB, heterozygote; BB, homozygote for minor allele; r^2 Pearson product-moment correlation coefficient between imputed and sequenced genotype.

SNP	Alleles	Concordance (imputed/sequenced)	r^2
rs28665337	AA	7/7	0.98
	AC	53/54	
	CC	142/141	
rs7449087	CC	106/106	0.81
	CT	76/56	
	TT	6/26	

Supplementary Table 5: Previously reported 8q24.21 cancer associated SNPs and LD with rs28665337.

Cancer	SNP	Reference	Previously Reported		Current ALL GWAS		LD with rs28665337	
			Risk Allele	*P* value	Risk Allele	*P* value	r^2	D′
Hodgkin's Lymphoma	rs2019960	1	C	6×10^{-10}	T	0.545	1.01×10^{-04}	-5.20×10^{-02}
Colorectal Cancer	rs6983267	2	G	5×10^{-14}	T	0.903	9.99×10^{-05}	-2.88×10^{-02}
Prostate Cancer	rs1447295	3	A	6×10^{-18}	C	0.523	3.97×10^{-05}	-6.73×10^{-03}
Prostate Cancer	rs12682344	3	G	5×10^{-12}	T	0.339	2.57×10^{-04}	-2.35×10^{-01}
Prostate Cancer (early onset)	rs10505477	4	A	9×10^{-9}	A	0.940	1.68×10^{-04}	-3.69×10^{-02}
Colorectal Cancer	rs10505477	5	T	8×10^{-13}	A	0.940	1.68×10^{-04}	-3.69×10^{-02}
Breast Cancer (early onset)	rs2392780	6		1×10^{-8}	A	0.892	7.82×10^{-04}	6.40×10^{-02}
Renal cell carcinoma	rs6470589	7	G	5×10^{-11}	G	0.510	1.59×10^{-04}	-3.77×10^{-02}
Bladder Cancer	rs9642880	8	T	4×10^{-38}	T	0.313	1.55×10^{-04}	3.16×10^{-02}
Chronic lymphocytic leukemia	rs2466035	9	C	2×10^{-8}	C	0.653	1.01×10^{-05}	-1.27×10^{-02}
Breast Cancer	rs13281615	10	G	1×10^{-17}	G	0.807	7.45×10^{-04}	-8.97×10^{-02}
Breast Cancer	rs11780156	10	T	3×10^{-11}	T	0.094	3.97×10^{-04}	-1.17×10^{-01}
Ovarian Cancer	rs10088218	11		1×10^{-17}	A	0.006	1.15×10^{-02}	1.16×10^{-01}
Prostate Cancer	rs6983561	12	C	4×10^{-13}	A	0.362	2.49×10^{-04}	-2.32×10^{-01}
Prostate Cancer	rs13254738	13	C	4×10^{-10}	A	0.639	7.52×10^{-04}	1.12×10^{-01}
Prostate Cancer	rs4242384	14	C	3×10^{-16}	A	0.419	1.07×10^{-04}	-1.11×10^{-02}
Prostate Cancer	rs1016343	14	T	4×10^{-10}	C	0.225	5.53×10^{-04}	-1.27×10^{-01}
Glioma	rs4295627	15	G	5×10^{-21}	T	0.097	1.55×10^{-04}	1.56×10^{-02}
Breast Cancer	rs1562430	16	A	3×10^{-11}	T	0.769	8.27×10^{-04}	6.56×10^{-02}
Prostate Cancer	rs1456315	17	A	2×10^{-29}	C	0.858	7.28×10^{-04}	1.10×10^{-01}
Prostate Cancer	rs7837688	17	T	1×10^{-25}	G	0.712	2.13×10^{-04}	-1.55×10^{-02}
Prostate Cancer	rs16902094	18	G	6×10^{-15}	A	0.856	1.39×10^{-05}	-2.44×10^{-02}
Prostate Cancer	rs16901979	18	A	3×10^{-14}	C	0.334	2.57×10^{-04}	-2.35×10^{-01}

Prostate Cancer	rs445114	18	T	5×10^{-10}	C	0.521	2.70x10^{-04}	-5.84x10^{-02}
Glioma	rs891835	19	G	8×10^{-11}	T	0.126	1.17x10^{-05}	-1.78x10^{-02}
Colorectal Cancer	rs7014346	20	A	9×10^{-26}	A	0.718	1.11x10^{-04}	-2.28x10^{-02}
Prostate Cancer	rs6983267	21	G	7×10^{-12}	T	0.903	9.99x10^{-05}	-2.88x10^{-02}
Glioma	rs55705857	22	A	$2X10^{-38}$	A	0.204	3.79x10^{-04}	2.91x10^{-02}
Diffuse large B-cell Lymphoma	rs13255292	23	T	$10X10^{-13}$			4.70x10-^{03}	1.39x10-^{01}
Diffuse large B-cell Lymphoma	rs4733601	23	A	$4X10^{-10}$			1.00x10-^{04}	3.01x10-^{02}

1) Frampton, M., et al. " Variation at 3p24.1 and 6q23.3 influences the risk of Hodgkin's lymphoma. " *Nature communications* **4** (2013).
2) Zhang, Ben, et al. "Large-scale genetic study in East Asians identifies six new loci associated with colorectal cancer risk." *Nature genetics* **46,** 533-542 (2014).
3) Knipe, Duleeka W., et al. "Genetic Variation in Prostate-Specific Antigen–Detected Prostate Cancer and the Effect of Control Selection on Genetic Association Studies." *Cancer Epidemiology Biomarkers & Prevention* **23,** 1356-1365 (2014).
4) Lange, Ethan M., et al. "Genome-wide association scan for variants associated with early-onset prostate cancer." *PloS one* **9** (2014).
5) Whiffin, Nicola, et al. "Identification of susceptibility loci for colorectal cancer in a genome-wide meta-analysis." *Human molecular genetics* **23**, 4729-4737 (2014).
6) Ahsan, Habibul, et al. "A Genome-wide association study of early-onset breast cancer identifies PFKM as a novel breast cancer gene and supports a common genetic spectrum for breast cancer at any age." *Cancer Epidemiology Biomarkers & Prevention* **23**, 658-669 (2014).
7) Gudmundsson, Julius, et al. "A common variant at 8q24. 21 is associated with renal cell cancer." *Nature communications* **4** (2013).
8) Figueroa, Jonine D., et al. "Genome-wide association study identifies multiple loci associated with bladder cancer risk." *Human molecular genetics* **23**, 1387-1398 (2014).
9) Berndt, Sonja I., et al. "Genome-wide association study identifies multiple risk loci for chronic lymphocytic leukemia." *Nature genetics* **45**, 868-876 (2013).
10) Michailidou, Kyriaki, et al. "Large-scale genotyping identifies 41 new loci associated with breast cancer risk." *Nature genetics* **45**, 353-361 (2013).
11) Pharoah, Paul DP, et al. "GWAS meta-analysis and replication identifies three new susceptibility loci for ovarian cancer." *Nature genetics* **45**, 362-370 (2013).

12) Xu, Jianfeng, et al. "Genome-wide association study in Chinese men identifies two new prostate cancer risk loci at 9q31. 2 and 19q13. 4." *Nature genetics* **44**, 1231-1235 (2012).
13) Cheng, Iona, et al. "Evaluating genetic risk for prostate cancer among Japanese and Latinos." *Cancer Epidemiology Biomarkers & Prevention* **21**, 2048-2058 (2012).
14) Schumacher, Fredrick R., et al. "Genome-wide association study identifies new prostate cancer susceptibility loci." *Human molecular genetics* **20**, 3867-3875 (2011).
15) Sanson, Marc, et al. "Chromosome 7p11. 2 (EGFR) variation influences glioma risk." *Human molecular genetics* ddr192 (2011).
16) Fletcher, Olivia, et al. "Novel breast cancer susceptibility locus at 9q31. 2: results of a genome-wide association study." *Journal of the National Cancer Institute* (2011).
17) Takata, Ryo, et al. "Genome-wide association study identifies five new susceptibility loci for prostate cancer in the Japanese population." *Nature genetics* **42**, 751-754 (2010).
18) Gudmundsson, Julius, et al. "Genome-wide association and replication studies identify four variants associated with prostate cancer susceptibility." *Nature genetics* **41**, 1122-1126 (2009).
19) Shete, Sanjay, et al. "Genome-wide association study identifies five susceptibility loci for glioma." *Nature genetics* 41.8 (2009): 899-904.
20) Tenesa, Albert, et al. "Genome-wide association scan identifies a colorectal cancer susceptibility locus on 11q23 and replicates risk loci at 8q24 and 18q21." *Nature genetics* **40**, 631-637 (2008).
21) Eeles, Rosalind A., et al. "Multiple newly identified loci associated with prostate cancer susceptibility." *Nature genetics* **40**, 316-321 (2008).
22) Enciso-Mora, Victor, et al. "Deciphering the 8q24. 21 association for glioma." *Human molecular genetics* (2013).
23) Cerhan J.R. et al. "Genome-wide association study identifies multiple susceptibility loci for diffuse large B cell lymphoma" *Nature Genetics* **46**, 1233-1238 (2014).

Supplementary Table 6: Relationship between newly discovered risk SNPs and event free survival and risk of relapse. *P* value associated with event free survival (EFS) and risk of relapse are shown; NA: data not available. Details of the statistical tests applicable are described in the methods section.

**rs28665337 (8q24.21)**		***P* value**	
**Trial**	**Subtype**	**EFS***	**Relapse***
UK ALL2003	BCP-ALL	0.90	0.90
	ETV6-RUNX1 positive	0.70	0.90
	ETV6-RUNX1 negative	0.90	0.80
UK ALL97-99	BCP-ALL	0.50	0.50
	ETV6-RUNX1 positive	0.80	0.80
	ETV6-RUNX1 negative	0.60	0.40
German ALL-BFM 2000	BCP-ALL	0.70	0.82
	ETV6-RUNX1 positive	NA[#]	NA
	ETV6-RUNX1 negative	NA	NA

**rs17481869 (2q22.3)**		***P* value**	
**Trial**	**Subtype**	**EFS***	**Relapse***
UK ALL2003	BCP-ALL	0.60	0.80
	ETV6-RUNX1 positive	0.50	0.40
	ETV6-RUNX1 negative	0.50	0.60
	MRD negative		0.30
	MRD positive		0.50
UK ALL97-99	BCP-ALL	0.30	0.50
	ETV6-RUNX1 positive	0.60	0.80
	ETV6-RUNX1 negative	0.40	0.90
	MRD negative		NA
	MRD positive		NA
German ALL-BFM 2000	BCP-ALL	0.22	0.03
	ETV6-RUNX1 positive	0.26	NA
	ETV6-RUNX1 negative	0.10	0.007
	MRD negative		0.4
	MRD positive		0.07

Supplementary Table 7: Results of the chromatin mark enrichment analysis of childhood ALL risk loci. Enrichment scores for histone marks of open chromatin H3K27ac, H3K4me1 and H3K4me3, using ChiP-seq data from six childhood ALL Bone marrow cell lines obtained from the Blue-print Epigenome data base. 14 cell-types from ENCODE, three AML cell bone marrow cell lines. Enrichment is measured as the fold – increase in ChiP-seq signal peaks at the 10 childhood ALL risk loci compared to a series of randomly generated null distributions.

**Childhood ALL cell line**	**Fold enrichment (*P*-value)**		
	**H3K27ac**	**H3K4me1**	**H3K4me3**
S0177HH1	6.84 ($1x10^{-4}$)	No data	5.78 ($2x10^{-4}$)
S0176JH1	6.15($1x10^{-4}$)	5.57($1x10^{-4}$)	3.76 ($3.1x10^{-3}$)
S01GQHH1	5.06 ($4.1x10^{-3}$)	4.43 ($5x10^{-4}$)	1.34 (0.49)
S01GRFH1	5.06 ($3 x10^{-4}$)	5.59 ($1x10^{-4}$)	5.89 ($1x10^{-4}$)
S0179DH1	4.9 ($6 x10^{-4}$)	4.17 ($5 x10^{-4}$)	No data
S017E3H1	4.54 ($1.1x10^{-3}$)	6.8 ($1x10^{-4}$)	3.67($3.7x10^{-3}$)

Supplementary Table 8: Genes that have P_{eQTL} <5x10^{-8} in the SMR analysis across the three publicly available datasets that are within 1MB of sentinel risk SNPs in each of the risk locus. SMR analysis for genes with at least one P_{eQTL} <5x10^{-8}. 38 genes were tested, stipulating a SMR significance threshold of P_{SMR} < 1.3x10^{-4}. Eight genes exceeded the P_{SMR} threshold, of which one passed the HEIDI test (P_{HEIDI}>0.05).

		P_{eQTL} <5x10^{-8}		
**Gene**	**GTEX**	**Muther_eQTL**	**Blood_Westra**	**Total**
OR6J1				0
OXA1L				0
SLC7A7		2.66x10^{-45}		1
MRPL52			5.04x10^{-75}	1
MMP14				0
LRP10			1.08x10^{-16}	1
REM2				0
RBM23		5.47x10^{-11}	1.63x10^{-48}	2
PRMT5-AS1				0
PRMT5	1.64x10^{-39}		7.5x10^{-99}	2
LOC101926933				0
HAUS4	3.86x10^{-27}	3.85x10^{-08}		2
MIR4707				0
AJUBA				0
C14orf93				0
PSMB5			3.59x10^{-21}	1
PSMB11				0
CDH24				0
ACIN1				0
C14orf119				0
CEBPE				0
SLC7A8				0
RNF212B				0
HOMEZ				0
PPP1R3E				0
BCL2L2-PABPN1				0
BCL2L2				0
PABPN1				0
SLC22A17				0
EFS				0
IL25				0
CMTM5				0
MYH6				0
MIR208A				0
MYH7				0
MHRT				0
MIR208B				0
NGDN				0
THTPA				0
ZFHX2				0
AP1G2	1.01x10^{-20}		7.1x10^{-23}	2
LOC102724814				0
JPH4				0

NTN4				0
LOC105369921				0
LINC02410				0
SNRPF			$2.22x10^{-25}$	1
CCDC38				0
AMDHD1	$1.23x10^{-31}$	$4.28x10^{-08}$	$1.4x10^{-127}$	3
HAL			$3.02x10^{-92}$	1
LTA4H	$1.00x10^{-10}$		$1.7x10^{-158}$	2
ELK3				0
CDK17				0
CFAP54				0
LINC00976				0
LINC00977				0
CCDC26				0
ZPBP				0
IKZF1				0
FIGNL1	$1.40x10^{-10}$	$8.98x10^{-12}$	$2.45x10^{-10}$	3
DDC				0
GRB10				0
MTAP			$1.65x10^{-29}$	1
CDKN2A				0
CDKN2B	$2.06x10^{-18}$		$3.03x10^{-17}$	2
TMEM26-AS1				0
C10orf107				0
ARDI5B				0
RTKN2	$1.45x10^{-09}$		$1.97x10^{-14}$	2
ZNF365			$2.67x10^{-79}$	1
LOC283045				0
ITIH5				0
ITIH2				0
KIN				0
TAF3				0
ATP5C1				0
GATA3-AS1				0
GATA3				0
LINC00708				0
EBNL1				0
COMMD3				0
SPAG6				0
PIP4K2A			$4.69x10^{-202}$	1
ARMC3				0
CHST15		$2.99x10^{-15}$		1
OAT		$7.41x10^{-18}$	$1.55x10^{-50}$	2
LHPP	$5.47x10^{-13}$	$7.85x10^{-11}$	$2.1x10^{-107}$	3
FAM53B			$3.12x10^{-13}$	1
EEF1AKMT2				0
FAM175B				0
ZRANB1		$4.58x10^{-41}$	0	2
CTBP2				0
			**Total**	**38**

Supplementary Table 9: Results from the SMR eQTL analysis of the combined summary GWAS datasets. P_{SMR}shows the summary *P* value from the analysis after filtering 5.2 $x10^{-5}$ from the SMR analysis. P_{HEIDI} shows the heterogeneity associated with each probe-SNP analysis (value <0.05 indicates significant heterogeneity). Two genes passing the P_{HEIDI} test (<0.05) are shown in bold.

Tissue	Chr	Probe ID	Gene	Probe bp	SNP	SNP bp	A1	A2	Freq (A1)
Whole blood (Westra *et al*)	9	ILMN_2376723	*CDKN2B*	22003161	rs3218002	22000841	A	G	0.118138
Whole blood (Westra *et al*)	9	ILMN_1723198	*CDKN2B*	22003948	rs1333034	22044122	C	T	0.085203
Whole blood (GTEX)	9	ENSG00000147883.9	*CDKN2B*	22002902	rs115574830	22059061	A	T	0.075736
**Whole blood (Westra *et al*)**	**10**	**ILMN_1704571**	***FAM53B***	**126308284**	**rs10901793**	**126324209**	**A**	**G**	**0.288942**
Whole blood (Westra *et al*)	7	ILMN_1778152	*FIGNL1*	50513148	rs4947641	50489038	T	C	0.449722
Whole blood (Westra *et al*)	7	ILMN_2389114	*FIGNL1*	50513147	rs4947641	50489038	T	C	0.449722
Whole blood (GTEX)	7	ENSG00000132436.7	*FIGNL1*	50511831	rs12719019	50476139	T	C	0.420286
**Whole blood (Westra *et al*)**	**10**	**ILMN_2152465**	***PIP5K2A***	**22826096**	**rs10764339**	**22867210**	**T**	**C**	**0.321241**
Muther eQTL	7	ILMN_2389114	*FIGNL1*	50511831	rs4947641	50489038	T	C	0.449722
Muther eQTL	7	ILMN_1778152	*FIGNL1*	50511831	rs4947641	50489038	T	C	0.449722

b_{GWAS}	SE_{GWAS}	P_{GWAS}	b_{eQTL}	SE_{eQTL}	P_{eQTL}	b_{XY}	*SE*	P_{SMR}	P_{HEIDI}
0.388681	0.0526	$1.43X10^{-13}$	-0.27024	0.0319977	$3.03X10^{-17}$	-1.43829	0.25854	$2.65X10^{-08}$	$2.31X10^{-03}$
0.418628	0.0521	$9.29X10^{-16}$	-0.20917	0.0316762	$4.02X10^{-11}$	-2.00138	0.392288	$3.36X10^{-07}$	$1.40X10^{-05}$
0.409314	0.056	$2.68X10^{-13}$	-0.54955	0.0627793	$2.07X10^{-18}$	-0.744822	0.132753	$2.02X10^{-08}$	$3.34X10^{-03}$
**0.14991**	**0.0348**	$\mathbf{1.65X10^{-05}}$	**-0.15159**	**0.0207964**	$\mathbf{3.12X10^{-13}}$	**-0.988948**	**0.266671**	$\mathbf{2.09X10^{-04}}$	$\mathbf{6.42X10^{-01}}$
-0.333782	0.0325	$1.05X10^{-24}$	0.168492	0.0192682	$2.24X10^{-18}$	-1.981	0.297637	$2.82X10^{-11}$	$2.38X10^{-07}$
-0.333782	0.0325	$1.05X10^{-24}$	0.122381	0.0193336	$2.45X10^{-10}$	-2.7274	0.50625	$7.15X10^{-08}$	$3.46X10^{-04}$
-0.341713	0.0324	$5.26X10^{-26}$	0.266226	0.0414959	$1.40X10^{-10}$	-1.28354	0.234171	$4.22X10^{-08}$	$6.62X10^{-10}$
**-0.182891**	**0.0335**	$\mathbf{4.60X10^{-08}}$	**-0.56413**	**0.0186001**	$\mathbf{4.69X10^{-202}}$	**0.3242**	**0.060268**	$\mathbf{7.48X10^{-08}}$	$\mathbf{3.21X10^{-01}}$
-0.333782	0.0325	$1.05X10^{-24}$	0.122986	0.018028	$8.98X10^{-12}$	-2.71398	0.47772	$1.34X10^{-08}$	$1.19X10^{-04}$
-0.333782	0.0325	$1.05X10^{-24}$	0.120998	0.0179542	$1.59X10^{-11}$	-2.75857	0.489709	$1.77X10^{-08}$	$4.74X10^{-04}$

Supplementary Table 10: eQTL analysis for genes within 2MB of rs28665337 and rs17481869 in whole blood using the GTEX portal online resources. eQTLs with *P*-values<0.05 are in bold.

Gene Symbol	Gencode Id	SNP	P-Value	Effect Size	T-Statistic	Standard Error	Tissue
**MYC**	**ENSG00000136997.10**	**rs28665337**	**0.00072**	**0.24**	**3.4**	**0.07**	**Whole Blood**
PCAT1	ENSG00000253438.2	rs28665337	0.067	-0.21	-1.8	0.11	Whole Blood
POU5F1B	ENSG00000212993.3	rs28665337	0.17	0.16	1.4	0.11	Whole Blood
CASC8	ENSG00000246228.2	rs28665337	0.21	0.16	1.3	0.13	Whole Blood
FAM49B	ENSG00000153310.14	rs28665337	0.29	-0.03	-1.1	0.028	Whole Blood
TMEM75	ENSG00000256655.1	rs28665337	0.38	0.11	0.89	0.13	Whole Blood
ASAP1	ENSG00000153317.10	rs28665337	0.59	0.026	0.54	0.047	Whole Blood
PVT1	ENSG00000249859.3	rs28665337	0.71	-0.027	-0.38	0.072	Whole Blood
GSDMC	ENSG00000147697.4	rs28665337	0.94	0.01	0.081	0.13	Whole Blood
**GTDC1**	**ENSG00000121964.10**	**rs17481869**	**0.037**	**-0.11**	**-2.1**	**0.052**	**Whole Blood**
ZEB2-AS1	ENSG00000238057.4	rs17481869	0.67	-0.049	-0.43	0.12	Whole Blood
ARHGAP15	ENSG00000075884.8	rs17481869	0.87	0.011	0.16	0.07	Whole Blood
ZEB2	ENSG00000169554.12	rs17481869	0.92	0.0053	0.099	0.053	Whole Blood

Supplementary Table 11: Individual variance in risk associated with childhood BCP-ALL SNPs. Chr: Chromosome; RAF: risk allele frequency. For each BCP-ALL risk loci the lead SNP is shown. Reference is provided if SNP has been described before. *P*-value from a fixed effects meta-analysis. * New loci discovered from this study. OR: Odds ratio; CI: confidence intervals. OR and CI are derived from current meta-analysis. Chr: chromosome. (BP): Base pair; % of total variance in risk to childhood ALL was tested under the following Standardised incidence ratios (SIR) of 3.2 (95% CI: 1.5-5.9) as per Kharazmi *et al.*[8]

									% of total variance in risk explained
**Chr**	**SNP**	**Locus (relevant gene)**	**Reference**	**Position(BP)**	**Risk Allele**	**RAF in controls**	**OR(95% CI)[c]**	***P*-value**	**3.2**
2	rs17481869	*2q22.3	This study	146124454	A	0.07	2.14 (1.64-3.35)	3.2×10^{-8}	4.21
7	rs10230978	7p12.2 (*IKZF1*)	This study	50477144	A	0.28	1.75 (1.63-1.88)	1.89×10^{-56}	7.06
8	rs28665337	*8q24.21	This study	130194104	A	0.12	1.33 (1.21-1.47)	3.86×10^{-9}	0.96
9	rs3731249	9p21.3 (*CDKN2A*)	(Vijayakrishnan *et al*, 2015 [7])	21970916	A	0.03	2.65 (2.22-3.17)	1.09×10^{-27}	3.09
9	rs3731217	9p21.3 (*CDKN2A*)	(Sherborne *et al*, 2010 [9])	21984661	G	0.85	0.70 (0.64-0.77)	7.24×10^{-14}	1.81
10	rs10821936	10q21.2 (*ARID5B*)	(Trevino *et al*, 2009 [10])	63723577	C	0.32	0.53 (0.50-0.57)	5.77×10^{-78}	9.81
10	rs3781093	10p14 (*GATA3*)	This study	8101927	C	0.14	1.34 (1.22-1.47)	1.18×10^{-10}	1.15
10	rs11013051	10p12.2 (*PIP4K2A*)	This study	22856279	C	0.77	0.79 (0.73-0.84)	5.92×10^{-12}	1.10
10	rs12779301	10q26.13 (*LHPP*)	This study	126292655	T	0.62	1.26 (1.18-1.35)	6.63×10^{-12}	1.41
14	rs2239630	14q11.2 (*CEBPE*)	This study	23589349	G	0.45	0.73 (0.69-0.78)	2.45×10^{-21}	2.74
12	rs1030137	12q23.1 (*ELK3*)	This study	96616051	C	0.29	1.21 (1.12-1.30)	1.35×10^{-7}	0.84
			**Overall %**						**34.18%**

1. Papaemmanuil, E., et al., *Loci on 7p12.2, 10q21.2 and 14q11.2 are associated with risk of childhood acute lymphoblastic leukemia.* Nat Genet, 2009. **41**(9): p. 1006-10.
2. Migliorini, G., et al., *Variation at 10p12.2 and 10p14 influences risk of childhood B-cell acute lymphoblastic leukemia and phenotype.* Blood, 2013. **122**(19): p. 3298-307.
3. Vijayakrishnan, J., et al., *A genome-wide association study identifies risk loci for childhood acute lymphoblastic leukemia at 10q26.13 and 12q23.1.* Leukemia, 2017. **31**(3): p. 573-579.
4. Westra, H.J., et al., *Systematic identification of trans eQTLs as putative drivers of known disease associations.* Nat Genet, 2013. **45**(10): p. 1238-43.
5. Martin, J.S., et al., *HUGIn: Hi-C Unifying Genomic Interrogator.* Bioinformatics, 2017.

6. Ay, F., T.L. Bailey, and W.S. Noble, *Statistical confidence estimation for Hi-C data reveals regulatory chromatin contacts.* Genome Res, 2014. **24**(6): p. 999-1011.
7. Vijayakrishnan, J., et al., *The 9p21.3 risk of childhood acute lymphoblastic leukaemia is explained by a rare high-impact variant in CDKN2A.* Sci Rep, 2015. **5**: p. 15065.
8. Kharazmi, E., et al., *Familial risks for childhood acute lymphocytic leukaemia in Sweden and Finland: far exceeding the effects of known germline variants.* Br J Haematol, 2012. **159**(5): p. 585-8.
9. Sherborne, A.L., et al., *Variation in CDKN2A at 9p21.3 influences childhood acute lymphoblastic leukemia risk.* Nat Genet, 2010. **42**(6): p. 492-4.
10. Trevino, L.R., et al., *Germline genomic variants associated with childhood acute lymphoblastic leukemia.* Nat Genet, 2009. **41**(9): p. 1001-5.

Supplementary Table 12: Oligonucleotides used in this study for Sanger sequencing and the PCR cycling conditions.

SNP Oligo name	Sequence
rs28665337_F	ACTCAGAATTTTGTCCATCAGCTC
rs28665337_F	GGGATTCAAGGAGAGAAAGGGA
rs7449087_F	GGAGTTGGGATTTTATAAGAGCAG
rs7449087_R	CCCTGTTTTCAGTGTTCCCTA

Cycling conditions for PCR amplification

Touch down PCR, QIAGEN Multiplex PCR reagents
Activation: 15 min 95°C
Denaturation: 30s 94°C
Annealing: 68-60°C
Extension: 90s 72°C
38 cycles
Final extension: 10 min 72°C

Supplementary Note 1

Acknowledgements

UK Bloodwise provided principal funding for the study (#13044). Support from Cancer Research UK (C1298/A8362, supported by the Bobby Moore Fund) is also acknowledged. Support from José Carreras Stiftung under project DJCLS R 13/19 is also acknowledged. This study makes use of data generated by the Wellcome Trust Case Control Consortium on the 1958 Birth Cohort and UK Blood service collection. A full list of the investigators who contributed to the generation of the data is available from www.wtccc.org.uk. Funding for the project was provided by the Wellcome Trust under award 076113. We thank the Bloodwise Childhood Leukaemia Cell Bank for access to samples. P. Thompson (Paediatric and Familial Cancer, Institute of Cancer Sciences, The University of Manchester) is funded by Children with Cancer. We are grateful to all the study subjects for their participation and their clinicians who contributed to the blood sample and data collection. The HNR study is supported by the Heinz Nixdorf Foundation (Germany). Additionally, the study is funded by the German Ministry of Education and Science and the German Research Council (DFG; Project SI 236/8-1, SI236/9-1, ER 155/6-1). The genotyping of the Illumina HumanOmni-1 Quad BeadChips of the HNR subjects was financed by the German Centre for Neurodegenerative Disorders (DZNE), Bonn. We are extremely grateful to all investigators who contributed to the generation of this dataset. We thank the High-Throughput Genomics Group at the Wellcome Trust Center for Human Genetics (funded by Wellcome Trust grant reference 090532/Z/09/Z) for the generation of the Sequencing data from the BCP-ALL cases. Genotyping of the PRACTICAL Oncoarray was funded by the US National Institutes of Health (NIH) [U19 CA 148537 for ELucidating Loci Involved in Prostate cancer SuscEptibility (ELLIPSE) project and X01HG007492 to the Center for Inherited Disease Research (CIDR) under contract number HHSN268201200008I]. Additional analytic support was provided by NIH NCI U01 CA188392 (PI: Schumacher). The PRACTICAL consortium was supported by Cancer Research UK Grants C5047/A7357, C1287/A10118, C1287/A16563, C5047/A3354, C5047/A10692, C16913/A6135, European Commission's Seventh Framework Programme grant agreement n° 223175 (HEALTH-F2-2009-223175), and The National Institute of Health (NIH) Cancer Post-Cancer GWAS initiative grant: No. 1 U19 CA 148537-01 (the GAME-ON initiative). We would also like to thank the following for funding support: The Institute of

Cancer Research and The Everyman Campaign, The Prostate Cancer Research Foundation, Prostate Research Campaign UK (now Prostate Action), The Orchid Cancer Appeal, The National Cancer Research Network UK, The National Cancer Research Institute (NCRI) UK. We are grateful for support of NIHR funding to the NIHR Biomedical Research Centre at The Institute of Cancer Research and The Royal Marsden NHS Foundation Trust. BCAC is funded by Cancer Research UK [C1287/A10118, C1287/A16563]. Genotyping of the OncoArray was funded by Genome Canada Grant GPH-129344, NIH Grant U19 CA148065, and Cancer UK Grant C1287/A16563. "The PERSPECTIVE project was supported by the Government of Canada through Genome Canada and the Canadian Institutes of Health Research (grant GPH-129344), the Ministère de l'Économie, Science et Innovation du Québec through Genome Québec, and the Quebec Breast Cancer Foundation. The BBCS is funded by Cancer Research UK and Breast Cancer Now and acknowledges NHS funding to the NIHR Biomedical Research Centre, and the National Cancer Research Network (NCRN). The coordination of EPIC is financially supported by the European Commission (DG-SANCO) and the International Agency for Research on Cancer. The national cohorts are supported by: Ligue Contre le Cancer, Institut Gustave Roussy, Mutuelle Générale de l'Education Nationale, Institut National de la Santé et de la Recherche Médicale (INSERM) (France); German Cancer Aid, German Cancer Research Center (DKFZ), Federal Ministry of Education and Research (BMBF) (Germany); the Hellenic Health Foundation, the Stavros Niarchos Foundation (Greece); Associazione Italiana per la Ricerca sul Cancro-AIRC-Italy and National Research Council (Italy); Dutch Ministry of Public Health, Welfare and Sports (VWS), Netherlands Cancer Registry (NKR), LK Research Funds, Dutch Prevention Funds, Dutch ZON (Zorg Onderzoek Nederland), World Cancer Research Fund (WCRF), Statistics Netherlands (The Netherlands); Health Research Fund (FIS), PI13/00061 to Granada, PI13/01162 to EPIC-Murcia, Regional Governments of Andalucía, Asturias, Basque Country, Murcia and Navarra, ISCIII RETIC (RD06/0020) (Spain); Cancer Research UK (14136 to EPIC-Norfolk; C570/A16491 and C8221/A19170 to EPIC-Oxford), Medical Research Council (1000143 to EPIC-Norfolk, MR/M012190/1 to EPIC-Oxford) (United Kingdom). SEARCH is funded by a programme grant from Cancer Research UK [C490/A10124] and supported by the UK National Institute for Health Research Biomedical Research Centre at the University of Cambridge. The UKBGS is funded by Breast Cancer Now and the Institute of Cancer Research (ICR), London. ICR acknowledges NHS funding to the NIHR Biomedical Research Centre. This study would not

have been possible without the contributions of the following: Manjeet K. Bolla, Qin Wang, Kyriaki Michailidou and Joe Dennis. We thank all the individuals who took part in these studies and all the researchers, clinicians, technicians and administrative staff who have enabled this work to be carried out. We thank the following for their contributions to this project: Jacques Simard, Peter Kraft, Craig Luccarini and the staff of the Centre for Genetic Epidemiology Laboratory; and Kimberly F. Doheny and the staff of the Center for Inherited Disease Research (CIDR) genotyping facility. We thank the participants and investigators of PERSPECTIVE, SEARCH, European Prospective Investigation into Cancer and Nutrition (EPIC). We thank Breast Cancer Now and the Institute of Cancer Research for support and funding of the Breakthrough Generations Study, and the study participants, study staff, and the doctors, nurses and other health care providers and health information sources who have contributed to the study.
