# Supplementary material for: Genome-wide association study identifies susceptibility loci for B-cell childhood acute lymphoblastic leukemia
Source: Nat Commun. 2018 Apr 9;9:1340. doi: 10.1038/s41467-018-03178-z (PMC5890276; doi:10.1038/s41467-018-03178-z)
Supplement: Supplementary file 3 — Description of Additional Supplementary Files [file 41467_2018_3178_MOESM3_ESM.pdf]

Description of Supplementary Files

File Name: Supplementary Data 1
Description: Functional annotations of BCP-ALL risk loci from the ENCODE HaploReg resource and eQTL data for risk loci from the Muther eQTL datasets.
